# Supplementary material for: Role of Two-Dimensional Ising Superconductivity in the Nonequilibrium Quasiparticle Spin-to-Charge Conversion Efficiency
Source: ACS Nano. 2021 Oct 1;15(10):16819–27. doi: 10.1021/acsnano.1c07192 (PMC8552497; doi:10.1021/acsnano.1c07192)
Supplement: Supplementary file 1 — nn1c07192_si_001.pdf [file nn1c07192_si_001.pdf]

Supplementary Information

# Role of Two-Dimensional Ising Superconductivity in the Non-Equilibrium Quasiparticle Spin-to-Charge Conversion Efficiency

Kun-Rok Jeon,[*†] Kyungjune Cho,[†] Anirban Chakraborty, Jae-Chun Jeon, Jiho Yoon, Hyeon Han, Jae-Keun Kim and Stuart S. P. Parkin[*]

*Max Planck Institute of Microstructure Physics, Weinberg 2, 06120 Halle (Saale), Germany*

[†]These authors contributed equally to this work.

[†]These authors contributed equally to this work.

*To whom correspondence should be addressed: jeonkunrok@gmail.com, stuart.parkin@halle-mpi.mpg.de

**This PDF file includes:**

Supplementary Text

Figs. *S1* to *S3*

References (*S1*-*S9*)

**S1. Dry transfer of 2H-$NbSe_2$ flakes onto magnon spin-transport devices.**

In this section, we describe the device fabrication process (Figure S1) with a focus on dry transfer techniques[S1] used.

1) As outlined in Methods (main text), we first fabricated standard magnon device structures consisting of a YIG channel and two Pt electrodes, along with outer Au/Ru leads for electrical contacts to the central 2H-$NbSe_2$ flake.

2),3) To dry-transfer the 2H-$NbSe_2$ flake, we utilized a polypropylene carbonate (PPC) coated dome shaped polydimethylsiloxane (PDMS) stamp. To make the dome shaped PDMS stamp, the PDMS was mixed with curing agent in a ratio of 1:10 (curing agent: PDMS) and baked at 100 °C on $SiO_2$/Si wafer. The baked PDMS was treated with oxygen plasma for 5 min and a PPC layer was spin-coated on the PDMS. Subsequently, the PPC coated PDMS was transferred onto the glass slide. After we aligned the PDMS stamp to a suitable 2H-$NbSe_2$ which was pre-transferred onto a $SiO_2$(300 nm)/Si substrate using a micromanipulator, the PDMS stamp was slowly approached to the 2H-$NbSe_2$ flake and heated up to 100 °C. Next, we cooled the stamp down to 40 °C and picked the 2H-$NbSe_2$ flake up.

4),5) The picked-up 2H-$NbSe_2$ flake was aligned to the pre-patterned magnon device structure by the micromanipulator and released at 100 °C.

6) The remained PPC residue was removed by acetone.

7) To prevent the unintentional contribution of iSHE from inner Au/Ru leads themselves to total voltage signals, we electrically isolate them from the active regime of magnon spin-transport by depositing an $Al_2O_3$ oxide in-between apart from the electric contact parts on top of the central/transferred 2H-$NbSe_2$ flake.

8) Finally, we defined the inner Au/Ru leads.

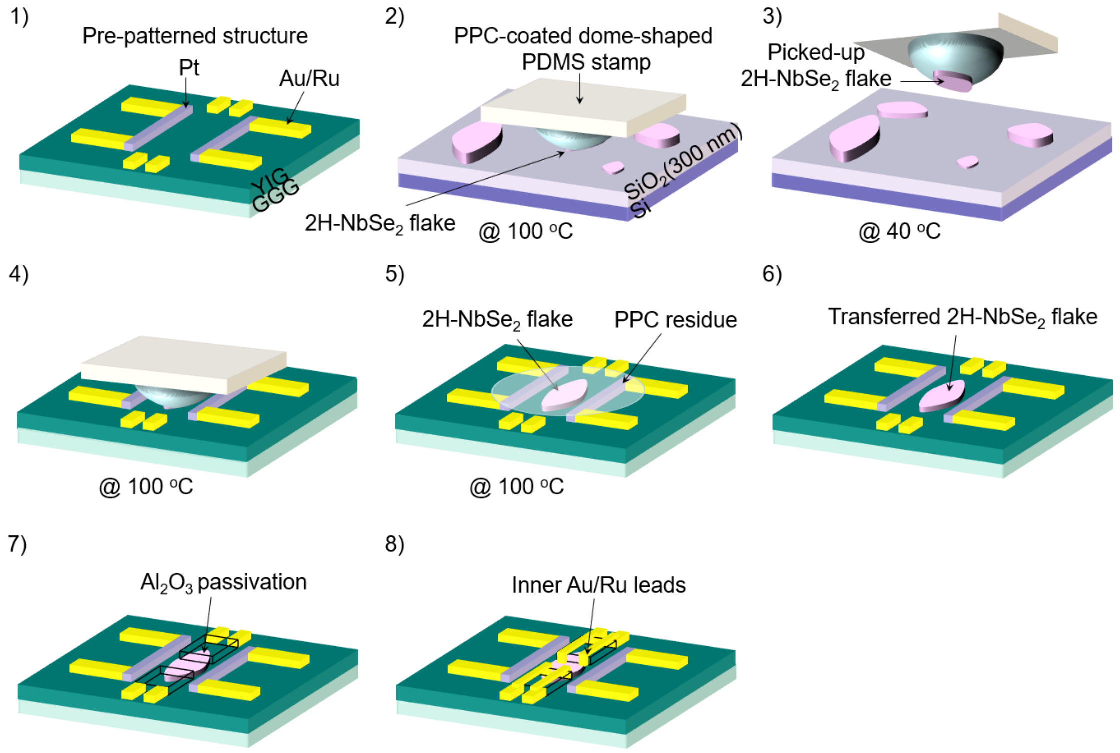


**Figure S1.** 1)-8) Schematic illustration of the device fabrication process flow.

**S2. Non-local spin signals detected by the Pt detector across $T_c$ of the 2H-NbSe$_2$ flake.**

For a given d.c. current $I_{dc}$ in the right Pt injector (Figure 2a,e), we simultaneously measure non-local voltages $[V_{nl}^{Pt}(\alpha),\ V_{nl}^{NbSe_2\ (or\ Nb)}(\alpha)]$ across the left Pt detector and the central 2H-NbSe$_2$ (or Nb) detector as a function of IP magnetic-field-angle $\alpha$. From this, we can confirm that magnon-carried spin currents propagate through a YIG channel to the left Pt detector, located farther away than the central 2H-NbSe$_2$ (or Nb) detector from the Pt injector, and also identify a sign of the spin-Hall angle $\theta_{SH}$ for each detector. Given $[\Delta V_{nl}^{th}]^{Pt} > 0$ (Figure S2c,S2g), $[\Delta V_{nl}^{th}]^{NbSe_2} < 0$ (Figure S2d) and $[\Delta V_{nl}^{th}]^{Nb} < 0$ (Figure S2h), and the fact that Pt detector is well known to have a positive $\theta_{SH}$ (> 0),[S2] one can conclude that the $\theta_{SH}$ signs for 2H-NbSe$_2$ and Nb[S3-S5] detectors are both negative (< 0) and the 4d heavy element Nb of the 2H-NbSe$_2$ dominates spin-to-charge conversion phenomena.

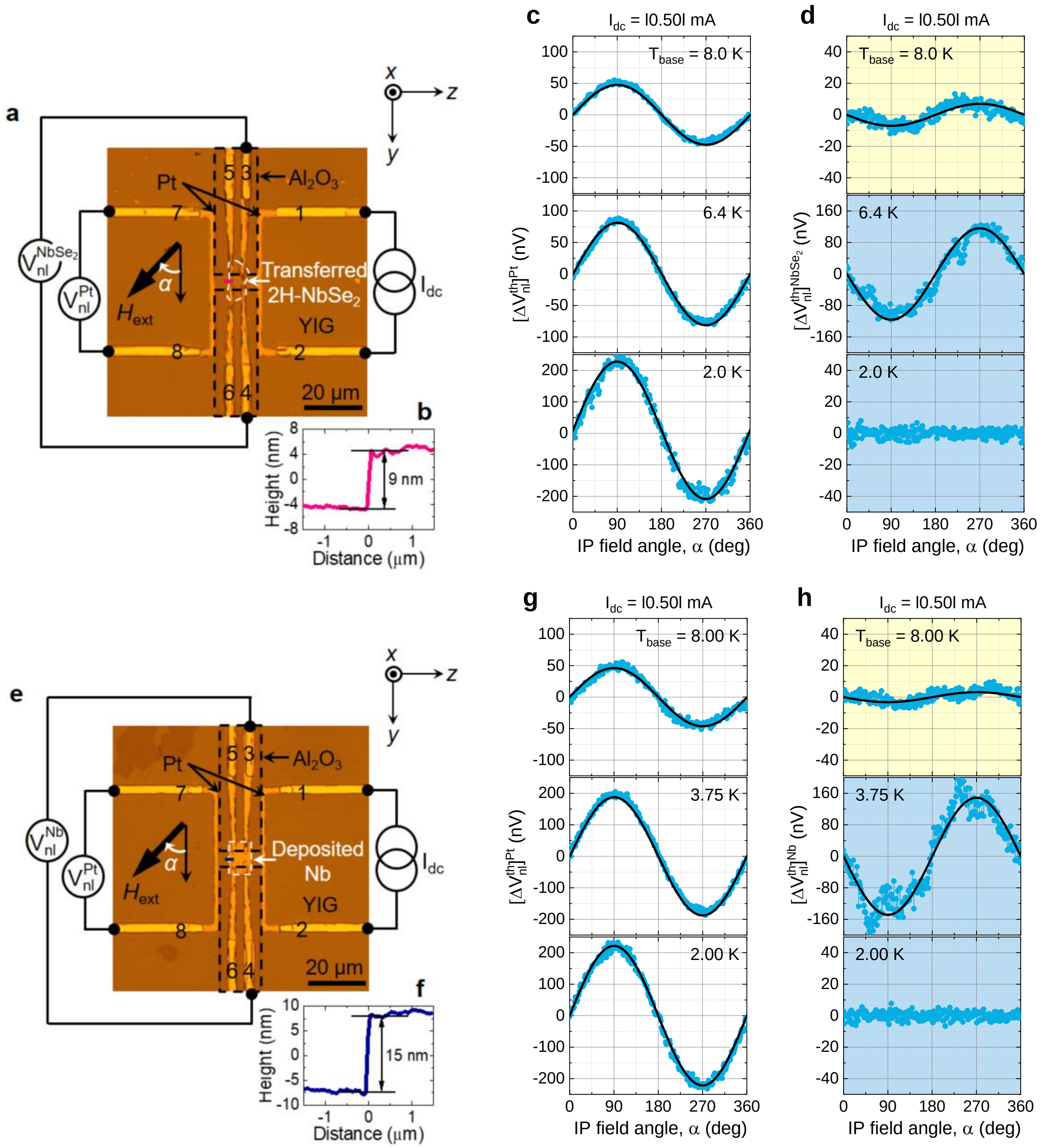


**Figure S2. a**) ptical micrograph of the $t_{NbSe_2}$ = 9 nm device. **b**) Atomic force microscopy (AFM) scan of the transferred 2H-NbSe$_2$ ($t_{NbSe_2}$ = 9 nm) flake. Thermally driven non-local voltages $[\Delta V_{nl}^{th}(\alpha)]^{Pt}$ (**c**) and $[\Delta V_{nl}^{th}(\alpha)]^{NbSe_2}$ (**d**) as a function of IP field angle $\alpha$ for the $t_{NbSe_2}$ = 9 nm device, taken at $I_{dc}$ = $|0.5|$ mA around $T_c$ of the 2H-NbSe$_2$. **e-h**) Data equivalent to **a-d** but for the $t_{Nb}$ = 15 nm reference device. Note that except for **c** and **g**, the others are also presented in the main text (Figure 1,3).

## S3. Transition-state enhancement of QP iSHE for the $t_{NbSe_2}$ = 2.5 nm device.

As discussed in the main text (Figure 4e,f), for the $t_{NbSe_2} < 3$ nm device, $T_c$ drops significantly and its transition width broadens anomalously (see Figure S3e). This causes sudden changes of the peak width and position of the transition-state QP iSHE (Figure 4f, main text) relative to the $t_{NbSe_2} > 3$ nm devices. These results can be ascribed to depressed superconductivity and smearing-out effect of QP DOS around the gap edge[S5,S6] due to enhanced thermal fluctuations at the 2D limit[S7,S8] ($t_{NbSe_2} < \xi^{\perp}_{NbSe_2} \approx 3$ nm), which inactivates the associated resonant absorption of magnon spin currents.[S4,S5,S9]

We also note that in the normal state ($T_{base} > T_c$), $\left[\Delta V_{nl}^{th}\right]^{NbSe_2}$ for $t_{NbSe_2}$ = 2.5 nm device (Figure S3d) is *at least 4 times* larger than $\left[\Delta V_{nl}^{th}\right]^{Nb}$ for $t_{Nb}$ = 15 nm device (Figure S2h), indicating high spin mixing conductance and spin transparency at the interface of our transferred 2H-NbSe$_2$ flake and YIG film.

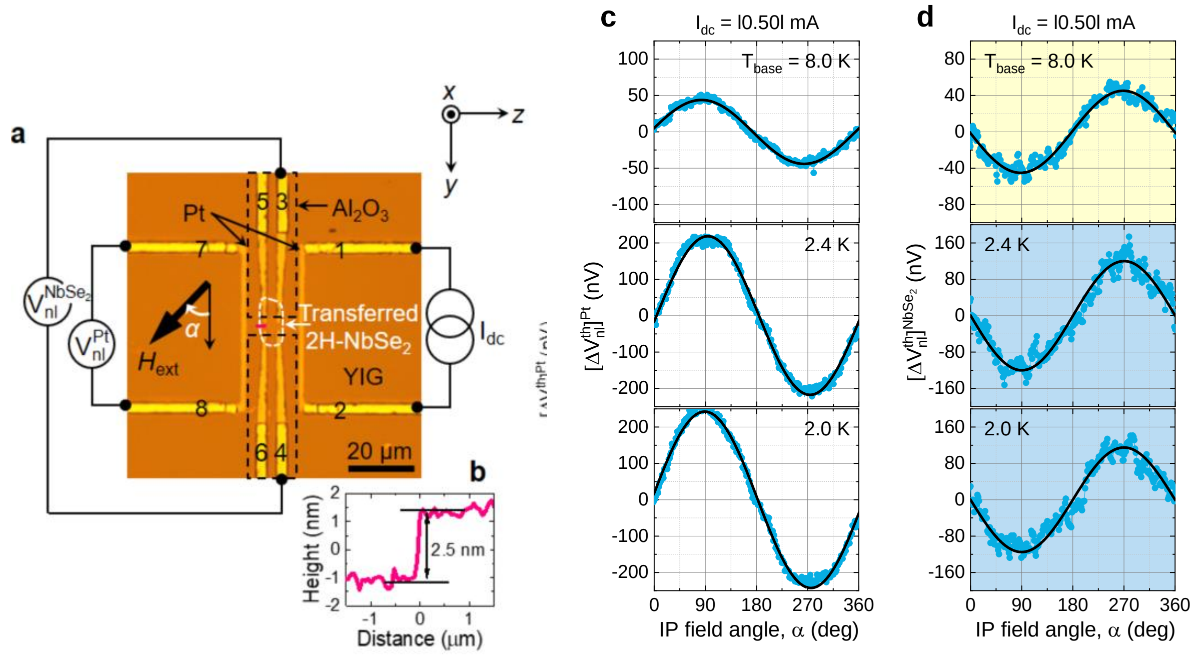

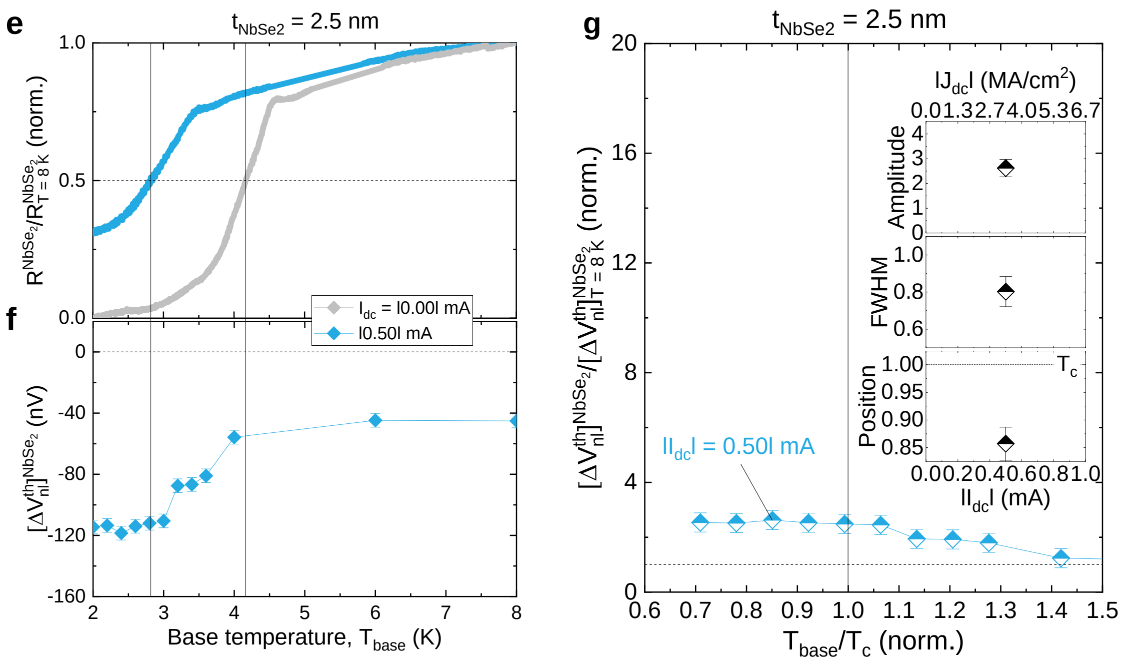


**Figure S3.** **a**) Optical micrograph of the $t_{NbSe_2}$ = 2.5 nm device. **b**) Atomic force microscopy (AFM) scan of the transferred 2H-NbSe2 ($t_{NbSe_2}$ = 2.5 nm) flake. Thermally driven non-local voltages $\left[\Delta V_{nl}^{th}(\alpha)\right]^{Pt}$ (**c**) and $\left[\Delta V_{nl}^{th}(\alpha)\right]^{NbSe_2}$ (**d**) as a function of IP field angle $\alpha$ for the $t_{NbSe_2}$ = 2.5 nm device, taken at $I_{dc}$ = $|0.5|$ mA around $T_c$ of the 2H-NbSe2. e, Normalized 2H-NbSe2 resistance $R^{NbSe_2}/R^{NbSe_2}_{T\ =\ 8\ K}$ *versus* $T_{base}$ plots for the $t_{NbSe_2}$ = 2.5 nm device, measured using a four-terminal current-voltage method (using leads 3, 4, 5, 6 in **a**) for $I_{dc}$ = $|0.0|$ and $|0.5|$ mA in the Pt injector. The critical temperature $T_c$ is defined as the point where $R^{NbSe_2} = 0.5R^{NbSe_2}_{T\ =\ 8\ K}$. **f**) Estimated magnitude of $\left[\Delta V_{nl}^{th}\right]^{NbSe_2}$ as a function of $T_{base}$ for the $t_{NbSe_2}$ = 2.5 nm device. **g**) $\left[\Delta V_{nl}^{th}\right]^{NbSe_2}/\left[\Delta V_{nl}^{th}\right]_{T\ =\ 8\ K}^{NbSe_2}$ *versus* $T_{base}/T_c$ plot for the $t_{NbSe_2}$ = 2.5 nm device. Each inset displays the $|I_{dc}|$ (or $|J_{dc}|$) dependence of the peak amplitude, width, and position.

## REFERENCES


[S1] R. Frisenda, E. Navarro-Moratalla, P. Gant, D. P. De Lara, P. Jarillo-Herrero, R. V. Gorbachev, A. Castellanos-Gomez, Recent Progress in the Assembly of Nanodevices and van der Waals Heterostructures by Deterministic Placement of 2D Materials. *Chem. Soc. Rev.* **2018**, *47*, 53.

[S2] J.-C. Rojas-Sánchez, N. Reyren, P. Laczkowski, W. Savero, J.-P. Attané, C. Deranlot, M. Jamet, J.-M. George, L. Vila, H. Jaffrès, Spin Pumping and Inverse Spin Hall Effect in Platinum: The Essential Role of Spin-Memory Loss at Metallic Interfaces. *Phys. Rev. Lett.* **2014**, *112*, 106602.

[S3] M. Morota, Y. Niimi, K. Ohnishi, D. H. Wei, T. Tanaka, H. Kontani, T. Kimura, Y. Otani, Indication of Intrinsic Spin Hall Effect in *4D* and *5D* Transition Metals. *Phys. Rev. B* **2011**, *83*, 174405.

[S4] K.-R. Jeon, C. Ciccarelli, H. Kurebayashi, J. Wunderlich, L. F. Cohen, S. Komori, J. W. A. Robinson, M. G. Blamire, Spin-Pumping-Induced Inverse Spin Hall Effect in Nb/$Ni_{80}Fe_{20}$ Bilayers and Its Strong Decay across the Superconducting Transition Temperature. *Phys. Rev. Appl*. **2018**, *10*, 014029.

[S5] K.-R. Jeon, J.-C. Jeon, X. Zhou, A. Migliorini, J. Yoon, S. S. P. Parkin. Giant Transition-State Quasiparticle Spin-Hall Effect in an Exchange-Spin-Split Superconductor Detected by Nonlocal Magnon Spin Transport. *ACS Nano* **2020**, *14*, 15874.

[S6] T. Kato, Y. Ohnuma, M. Matsuo, J. Rech, T. Jonckheere, T. Martin, Microscopic Theory of Spin Transport at the Interface between a Superconductor and a Ferromagnetic Insulator. *Phys. Rev. B* **2019**, *99*, 144411.

[S7] X. Xi, Z. Wang, W. Zhao, J.-H. Park, K. T. Law, H. Berger, L. Forró, J. Shan, K. F. Mak, Ising Pairing in Superconducting $NbSe_2$ Atomic Layers. *Nat. Phys.* **2016**, *12*, 139.

[S8] M. Tinkham, Introduction to Superconductivity, 2nd ed.; McGraw-Hill: New York **1996**.

[S9] M. Umeda, Y. Shiomi, T. Kikkawa, T. Niizeki, J. Lustikova, S. Takahashi, E. Saitoh, Spin-Current Coherence Peak in Superconductor/Magnet Junctions. *Appl. Phys. Lett.* **2018**, *112*, 232601.
